# How can we optimize the long-term outcome in children with intracranial cavernous malformations? A single-center experience of 61 cases

**DOI:** 10.1007/s10143-022-01823-2

**Published:** 2022-06-09

**Authors:** Dorian Hirschmann, Thomas Czech, Karl Roessler, Paul Krachsberger, Shivam Paliwal, Olga Ciobanu-Caraus, Anna Cho, Andreas Peyrl, Martha Feucht, Josa Maria Frischer, Christian Dorfer

**Affiliations:** 1 Department of Neurosurgery, Medical University of Vienna, Vienna, Austria; 2 Department of Pediatrics and Adolescent Medicine, Medical University of Vienna, Vienna, Austria; 3 Center for Rare and Complex Epilepsies, ERN EpiCARE. Department of Pediatrics and Adolescent Medicine, Medical University of Vienna, Vienna, Austria

**Keywords:** Pediatric cavernous malformation, Brainstem, Lobar, Conservative, Seizures

## Abstract

The objective is to provide a treatment algorithm for pediatric patients with intracranial cavernous malformations (CMs) based on our experience. Patients < 18 years of age who were treated either surgically or conservatively at the authors’ institution between 1982 and 2019 were retrospectively evaluated. A total of 61 pediatric patients were treated at the authors’ institution: 39 with lobar CMs; 18 with deep CMs, including 12 in the brainstem and 6 in the basal ganglia; and 4 with CMs in the cerebellar hemispheres. Forty-two patients underwent surgery, and 19 were treated conservatively. The median follow-up time was 65 months (1–356 months). In surgically treated patients, lesions were larger (2.4 cm vs 0.9 cm, *p* < 0.001). In patients with lobar CMs, seizures were more common (72% vs 21%, *p* = 0.003) in the surgery group than in conservatively managed patients. In deep CMs, modified Rankin scale (mRS) was higher (4 vs 1, *p* = 0.003) in the surgery group than in conservatively treated patients. At the time of last follow-up, no differences in Wieser outcome class I were seen (86% vs 67%) in lobar CMs, and mRS scores had aligned between the treatment groups in deep CMs (1 vs 0). We encountered no new permanent neurological deficit at time of last follow-up. We propose a treatment algorithm according to lesion location and size, burden of symptoms, epilepsy workup, and further clinical course during observation. A conservative management is safe in pediatric patients with asymptomatic CMs. Gross total resection should be the aim in patients with symptomatic lobar CMs. A less aggressive approach with subtotal resection, when required to prevent neurological compromise, sustainably improves neurological outcome in patients with deep CMs.

## Introduction

Cavernous malformations (CMs) are angiographic occult vascular lesions with a prevalence of 0.1–0.5% in the population [7, 22, 27]. Pediatric cerebral cavernous malformations account for approximately 25% of all cases and are a major cause of brain hemorrhage in children [11, 26]. Since the introduction of magnetic resonance imaging (MRI), a rise in the incidence of CMs has been reported, appearing as “mulberry-like” lesions in the brain [2, 10, 21]. The spectrum of the clinical presentation encompasses no symptoms in cases with incidental diagnosis of chronic symptoms in cases with epileptic seizures, or acute severe neurologic impairments caused by hemorrhage [9]. This wide spectrum of disease manifestation is reflected by a variety of potential treatment approaches [20].

The counseling of the patients’ parents for the optimal treatment approach to achieve the best long-term outcome in these children is challenging. There is a frequent and profound equipoise between a surgical or conservative management, aggressive or less aggressive surgery, and immediate or delayed intervention [3]. The clinical and imaging characteristics are crucial for decision-making, which, however, is complicated by two factors: first, CMs are histologically benign lesions without the potential for malignant transformation or extensive growth and, second, in contrast to shunting vascular lesions and aneurysms, the risk for hemorrhage is not necessarily a reason to decide for a surgical resection as (i) hemorrhage of CMs in non-eloquent areas cannot be compared with that of lesions in the brainstem, (ii) the sequelae of a bleeding is less dramatic and disabling compared with other vascular lesions, and (iii) the overall risk for a clinically relevant hemorrhage is considerably lower than in shunting vascular lesions or aneurysms [6, 15, 18].

The wealth of data on the clinical management of pediatric CMs is contrasted by the sparse number of studies reporting on their experience with pediatric CMs in the light of lesion location and natural history of conservatively treated CMs [2, 4, 17, 19, 24, 25, 32]. Here, we present our experience in treating this vulnerable patient population and suggest a treatment algorithm that may aid clinicians in determining the most favorable approach and their counseling of these children’s parents.

## Materials and methods

### Study design

Patients < 18 years of age at time of diagnosis (conservative group) or at time of surgery (surgery group) who were treated at our institution between 1982 and 2019 were included in this retrospective study. Histological confirmation of the diagnosis was mandatory in patients who underwent surgery, while a typical radiological appearance of a mulberry-like lesion with a rim of signal loss due to hemosiderin was key in conservatively managed patients. Superficial lesion location was defined as a maximum distance of 2 mm from the surface of the brain based on T1-weighted MRI images. Demographic patient data, radiological features, and clinical pre- and postoperative data were retrospectively extracted from clinical records. Radiology reports were reviewed to assess radiological features in cases of missing imaging data. Patients who were lost to follow-up were excluded from the outcome analyses.

### Treatment strategy

While the treatment decisions were made on an individual case-by-case basis, the management of these children shared some principal concepts that did not change during the course of the study period. Asymptomatic patients were primarily treated conservatively. If patients suffered from chronic pharmaco-resistant epileptic seizures, a formal epilepsy workup was performed, which included at least video-EEG monitoring, dedicated MR imaging protocol for epilepsy, and neurologic and psychological evaluation. In case the first seizure event was associated with diagnosis of a single CM and referral to our center, the decision for surgery may have been made without a complete presurgical epilepsy workup. In patients with deep lesions (i.e., brainstem and basal ganglia), immediate surgery was performed only if patients were unstable and further delay could not be justified. Otherwise, patients were initially observed, and surgery was only initialized in case of further deterioration or lack of clinical improvement. In case of a CM involving eloquent cortical regions, additional functional imaging such as diffusion tensor imaging (DTI) and functional MRI were performed to visualize motor and language cortical areas and pathways. Furthermore, and specifically in case of brainstem location, comprehensive intraoperative monitoring techniques, if required, and standard neuro-navigation were used to optimize the surgical trajectory. In general, the aim of surgery for CM was gross total resection with resection of the hemosiderin rim in lobar lesion in case it was confined to the CM. No attempt was made for a complete removal in case of extended hemosiderin deposits in the white matter. The goal of gross total removal was abandoned in case the risk for surgical morbidity outweighed the benefit of achieving a complete resection. Hence, decompression of adjacent structures by partial resection and/or hematoma evacuation may be performed in symptomatic patients with a CM in a highly eloquent region such as brain stem or basal ganglia. A reoperation was performed in case gross-total resection was not achieved initially and patients suffered from recurring symptoms.

Patients with multiple lesions in terms of a familial CM disease were closely followed by regular MRI examinations. In these patients, surgery was mainly performed in case of neurological deficit due to mass effect of one of the lesions. Epileptic seizures, however, were not a main indication for surgery in patients with familial CM disease. Surgical resection was performed only in exceptional cases of both high burden of disease and if a clear epileptogenic focus in the region of a single lesion was confirmed during epilepsy workup.

### Outcome evaluation

The primary outcome parameters of this study were the functional outcome and seizure control rate. Functional outcome was assessed by the modified Rankin Scale (mRS) and seizure outcome according to Wieser classification [29, 31]. Immediate and follow-up MRI data were assessed for the extent of resection and recurring disease. The prospective hemorrhage rate was used as the secondary outcome parameter. Consistent with prior studies, hemorrhagic events were defined as overt bleedings, which could be verified based on imaging studies and were accompanied by neurological symptoms [13, 15, 28].

### Data analysis

Data are presented as counts and percentages or as median and range. Categorical variables were analyzed by chi-square test. Mann–Whitney *U* test was used to identify differences between metric variables. Differences between pre- and postoperative mRS scores were assessed using the Wilcoxon signed-rank test. The prospective hemorrhage rate was calculated as number of hemorrhages divided by the cumulative follow-up duration in patient-years. Actuarial hemorrhage rates were calculated by Kaplan–Meier survival analysis and compared with Breslow-Test. Statistical analyses were conducted using IBM SPSS Statistics for Windows (Version 24 Armonk, NY: IBM Corp.) with the significance level set to *α* = 0.05. A central death register comparison was performed via “Statistic Austria”.

## Results

### Study population

Between 1982 and 2019, a total of 64 pediatric patients were treated for an intracranial CM at the Department of Neurosurgery, Medical University of Vienna. Two patients who underwent stereotactic radiosurgery only and one patient receiving radiotherapy 4 years before surgery were excluded. Among the 61 patients evaluated, 42 underwent microsurgery and 19 were managed conservatively. Baseline characteristics of both groups are presented in Table 1. There was no difference in gender distribution between the treatment groups, and there was a trend towards higher age in patients in the conservative group. Patients in the surgery group presented significantly more often with signs of acute hemorrhage (*p* = 0.001). Furthermore, lesions in the surgery group were significantly larger than in the conservative group (2.4 cm vs 0.9 cm median maximum diameter, *p* < 0.001). In accordance with these findings, the distribution of mRS at diagnosis differed between the treatment groups (*p* = 0.001), suggesting a higher burden of symptoms in the surgery group, although the median mRS at diagnosis was 1 in both groups. Symptoms at diagnosis encompassed various neurological deficits such as seizures, headache, hemiparesis, cranial nerve palsy, gait disorder, vertigo, and reduced state of consciousness in both treatment groups.

**Table 1:** Baseline characteristics data of the whole cohort of 61 patients

	Surgery (*n* = 42)	Conservative (*n* = 19)	*p*-value
Male sex (%)	25 (60%)	9 (47%)	0.415
Median age at time of diagnosis, years (min–max)	7 (1–17)	13 (1–17)	0.134
Hemorrhage at time of diagnosis (%)	32 (76%)	6 (32%)	***p* = 0.001**
Number of lesions			
Single CM	28 (67%)	10 (53%)	*p* = 0.394
Multiple CMs	14 (33%)	9 (47%)	
Familial CM disease	11 (26%)	5 (26%)	*p* = 1.000
Median lesion diameter (cm)	2.4 (0.7–5.8)	0.9 (0.5–2.6)	***p* < 0.001**
Median lesion volume (cm^3^)	4.0 (0.1–64.6)	0.3 (0.1–4.1)	***p* < 0.001**
Symptoms at diagnosis			
Seizures	19 (45%)	5 (26%)	*p* = 0.258
Headache	11 (26%)	8 (42%)	*p* = 0.243
Paresis	9 (21%)	1 (5%)	*p* = 0.151
Other neurological deficit	19 (45%)	4 (21%)	*p* = 0.091
None	1 (2%)	5 (26%)	***p* = 0.009**
mRS at time of diagnosis	1 (0–5)	1 (0–2)	***p* < 0.001**
Incidental finding	0	5 (26%)	***p* = 0.002**
Location			
Supratentorial	29 (69%)	16 (84%)	*p* = 0.346
Infratentorial	13 (31%)	3 (16%)	
Location detailed			
Lobes	25 (59%)	14 (73%)	*p* = 0.644
Pons	10 (24%)	2 (11%)	
Cerebellum	3 (7%)	1 (5%)	
Basal ganglia/thalamus/mid brain	4 (10%)	2 (11%)	
Depth			
Superficial	31 (74%)	8 (42%)	***p* = 0.031**
Profound	11 (26%)	10 (53%)	
Median time to surgery (days)	16 (1–1181)	–	–
Number of surgeries			
1	34 (82%)	–	–
2	6 (14%)		
3	1 (2%)		
6*	1 (2%)		
Postoperative complications	4 (10%)	–	–

Table 1 summarizes baseline characteristics of the whole study cohort which includes 42 patients who underwent surgery and 19 conservatively treated patients. Surgical data for the surgery group is included. Patients who underwent surgery presented more frequently with acute hemorrhage and higher mRS score. Lesions in the surgery group were larger but more often located superficially. *One patient underwent 4-time surgery for recurrent pons CM as well as 2-time surgery for recurrent CM of the right central region. Bold font indicates significance.

### Surgical treatment

Most patients underwent single (34/42, 82%) or two-time (6/42, 14%) surgery. Of 8 patients with more than one operation, 3 had a newly diagnosed CM in a different location and 5 patients underwent multiple surgeries to treat the same lesion. Of note, one patient underwent 6 surgeries, 4 of which were for a recurrent CM in the pons while 2 were for a recurrent CM of the right central region. Given the high probability of generating new neurological deficits when attempting aggressive removal, CM residuals were intentionally left in place in all cases of subtotal resection. The overall rate of complications was 10% (4/42) and included one subgaleal pseudomeningocele, one epidural hematoma, and one subdural hematoma, all of which resolved after revision surgery. Furthermore, there was one case of temporary postoperative neurological worsening. Complication rates for the subgroups are described in more detail below. No deaths occurred during the observation period.

### Overall clinical outcome

**Fig. 1:**
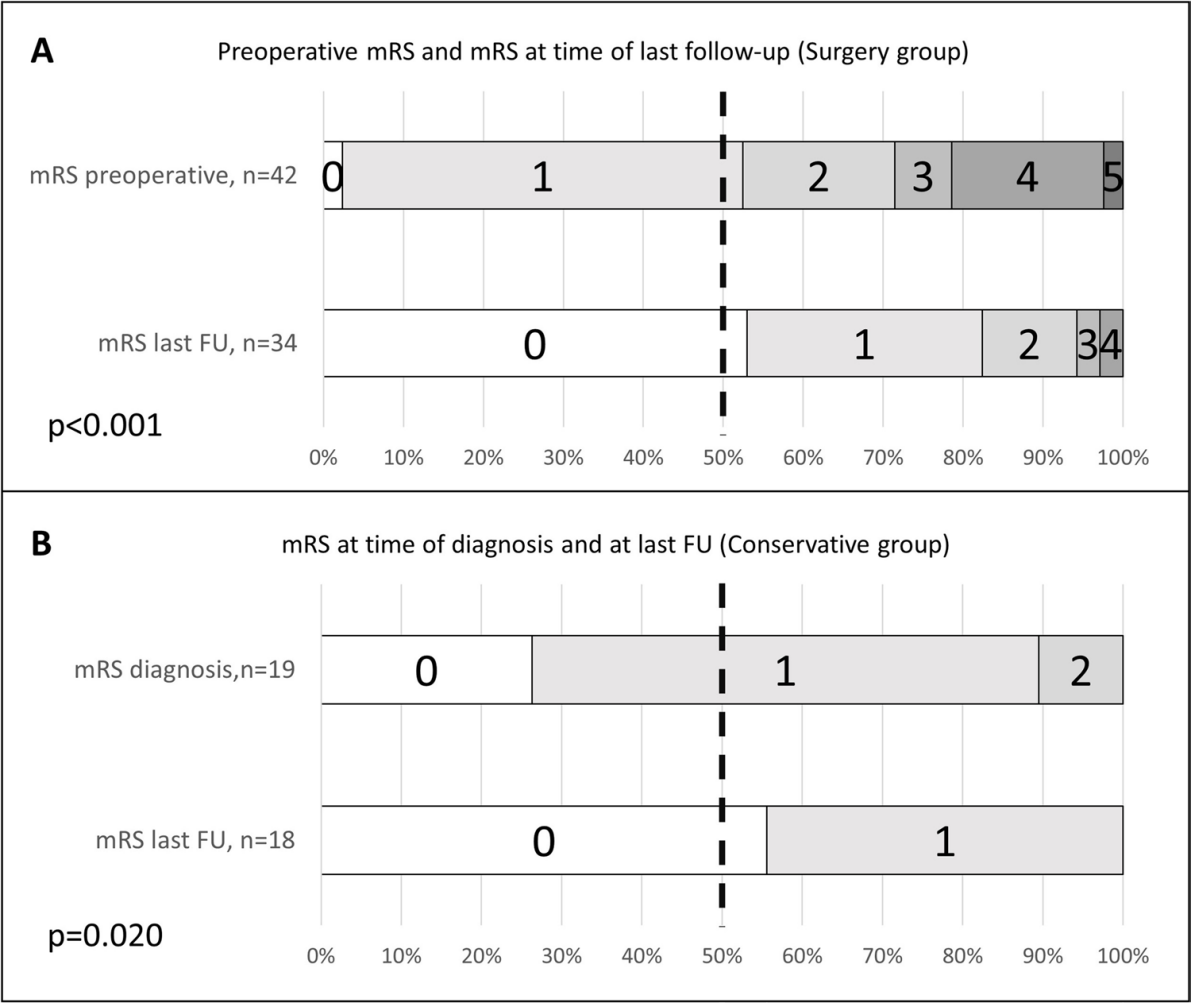
**A**, **B** The median mRS improved during follow-up from 1 to 0 in the surgery group (*p* < 0.001) as well as in the conservative group (*p* = 0.02)

The median time of follow-up from diagnosis (conservative group) and surgery (surgery group) was 59.5 (1–307 months) and 87 months (3–356 months), respectively. Time to surgery after diagnosis was 16 days (1–1181). Reasons for delayed initiation of surgical treatment after diagnosis were a low burden of symptoms with aggravation over time and initial anti-epileptic drug (AED) therapy of CM-associated epilepsy to determine pharmaco-resistance. A minimum clinical follow-up of 1 month was available in 85% (52/61) of patients, and long-term follow-up (≥ 1 year) was available in 77% (47/61) of patients. As shown in Fig. 1A and B, the median mRS improved during follow-up from 1 to 0 in the surgery group (*p* < 0.001) as well as in the conservative group (*p* = 0.02). Of note, the preoperative higher median mRS score among patients in the surgery group correlated with that of the conservative group during follow-up. Thus, no difference between median mRS at last follow-up was seen between the treatment groups (*p* = 0.639). In the surgery group, 94% (32/34) of patients had a favorable outcome versus 100% (18/18) in conservatively managed patients (mRS 0–2). Most common neurological symptoms at last follow-up included hemiparesis, headache, cranial nerve palsy, gait disorder, and vertigo. Detailed information about clinical status is shown in Table 1.

Patients were divided into two groups to account for the differences based on CM location: (i) lobar lesions and (ii) deep lesions, including brainstem and basal ganglia location. Of note, 4 patients were included in the overall analysis but excluded from subgroup analyses due to CM locality in the cerebellum without involvement of the brainstem.

### Lobar lesions

**Fig. 2:**
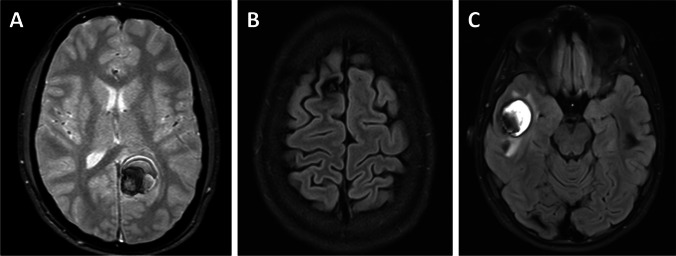
Patients presented in diverse clinical conditions with acute neurological symptoms other than seizures (**A**), epileptogenic lesions (**B**), and asymptomatic CMs (**C**)

A total of 64% (39/61) patients had a lobar CM. Patients presented in diverse clinical conditions with acute neurological symptoms other than seizures (Fig. 2A), epileptogenic lesions (Fig. 2B), and asymptomatic CMs (Fig. 2C). Of the whole subgroup, 64% (25/39) of patients underwent surgery, while 36% (14/39) were managed conservatively. Pre- and postoperative data about patients with lobar CMs are shown in Table 2. As expected, there was a clear difference in the surgery group based on surgical accessibility and size of the lesions. Accordingly, CMs in the surgery group were located superficially in 92% (23/25) of patients versus 57% (8/14) in the conservative group and were significantly larger (2.0 vs 1.1 mm maximum diameter, *p* = 0.002). The median mRS score at diagnosis was 1 (0–2) in both treatment groups and was statistically not different. Nevertheless, epileptic seizures at time of diagnosis occurred significantly more frequently in patients who underwent surgery than in conservatively treated patients (72% vs 21%, *p* = 0.003). These observations suggest that seizures were the leading symptom in surgically treated patients, whereas asymptomatic patients were more common in the conservative group (*p* = 0.016). This difference, however, was no longer seen at time of last follow-up where the proportion of patients classified as Wieser class I was 86% (12/14) in the surgery group and 67% (2/3) in the conservative group. In the surgery group, 2 patients received epilepsy surgery after CM resection due to persisting seizures, where seizures recurred 1 year and 3.5 years after initial CM resection, respectively. Epilepsy workup was performed using video EEG monitoring in both cases, including the use of subdural strip electrodes in one case. In both patients, the epileptogenic zone adjacent to the field of resection was identified. Both patients were seizure-free after resection of the epileptogenic focus. Two surgically treated patients were classified as Wieser III at last follow-up. One patient had only mild focal somatosensory seizures, and one patient suffered from a secondary generalized seizure 3 years after surgery. However, no further clinical information was available thereafter. In the conservative group, one patient who was classified as Wieser III at last follow-up experienced a generalized seizure 1 year after cessation of AEDs, which was then re-established. Reasons for conservative treatment of patients with seizures were deep lesion location adjacent to the motor cortex and presence of multiple lesions without clear identification of an epileptogenic focus during epilepsy workup. In each treatment group, one patient suffered from seizures at time of last follow-up but had no history of seizures at diagnosis or preoperative seizures and, thus, was categorized as having new-onset seizures. The patient in the surgery group underwent epilepsy surgery and the patient in the conservative group received AEDs. Functional outcome analysis revealed that the median mRS score at time of last follow-up improved to 0 in both treatment groups.

**Table 2:** Lobar CMs

Baseline	Surgery (*n* = 25)	Conservative (*n* = 14)	*p*-value
Location			
Frontal	6 (24%)	3 (21%)	–
Temporal	5 (20%)	2 (14%)	
Occipital	5 (20%)	2 (14%)	
Parietal	5 (20%)	5 (36%)	
Central	2 (8%)	2 (14%)	
Cingulum	2 (8%)	0	
Hemorrhage at time of diagnosis (%)	15 (60%)	5 (36%)	*p* = 0.191
Seizures at diagnosis	18 (72%)	3 (21%)	***p* = 0.003**
Free of symptoms	1 (4%)	5 (36%)	***p* = 0.016**
Median mRS at time of diagnosis (min–max)	1 (0–2)	1 (0–2)	0.141
Median maximum lesion diameter (cm)	2.0 (0.7–5.8)	1.1 (0.5–2.6)	***p* = 0.002**
Depth			
Superficial	23 (92%)	8 (57%)	***p* = 0.016**
Profound	2 (8%)	6 (43%)	
Number of interventions			
1	21		
2	4	n.a	**–**
Postoperative complication	2 (8%)	n.a	**–**
Gross-total resection	24 (96%)	n.a	**–**
Outcome	Surgery (*n* = 21)	Conservative (*n* = 13)	
Median time of follow-up (months)	64.0 (3–322)	58.0 (5–307)	*p* = 0.501
Median mRS at time of last follow-up	0 (0–2)	0 (0–1)	*p* = 0.545
Hemorrhage during follow-up	0	1 (8%)	*p* = 0.382
Seizures at last follow-up	5 (24%)	2 (15%)	*p* = 0.345
Seizure Outcome	Surgery (*n* = 14)	Conservative (*n* = 3)	
Wieser class at time of last follow-up			
I	12 (86%)	2 (67%)	*p* = 0.465
III	2 (14%)	1 (33%)	

Table 2 shows baseline characteristics for patients with CMs located in the lobes of the telencephalon grouped according to surgical (*n* = 25) and conservative (*n* = 14) treatment. Outcome data was available for 21 patients of the surgery group and for 13 patients of the conservative group. Wieser outcome classes are given for patients who had suffered from preoperative seizures and for whom outcome data was available (*n* = 14 and *n* = 3). Bold font indicates significance.

A total of 8% (2/25) of patients with lobar CMs suffered from postoperative complications. One patient experienced an epidural hematoma after CM resection, needing urgent evacuation without any neurological sequelae. Another patient had to undergo evacuation of an epidural hematoma after second surgery. No new permanent neurological deficits were recorded in the lobar CM group. Gross-total CM resection (GTR) was achieved in 96% (24/25) of patients. GTR was avoided in one patient due to location adjacent to the caudate nucleus. Except for the three patients who underwent subsequent epilepsy surgery after CM resection and one patient who underwent second surgery due to an additional cerebellar CM, the remaining 21 patients underwent single surgery.

### Deep lesions

**Fig. 3:**
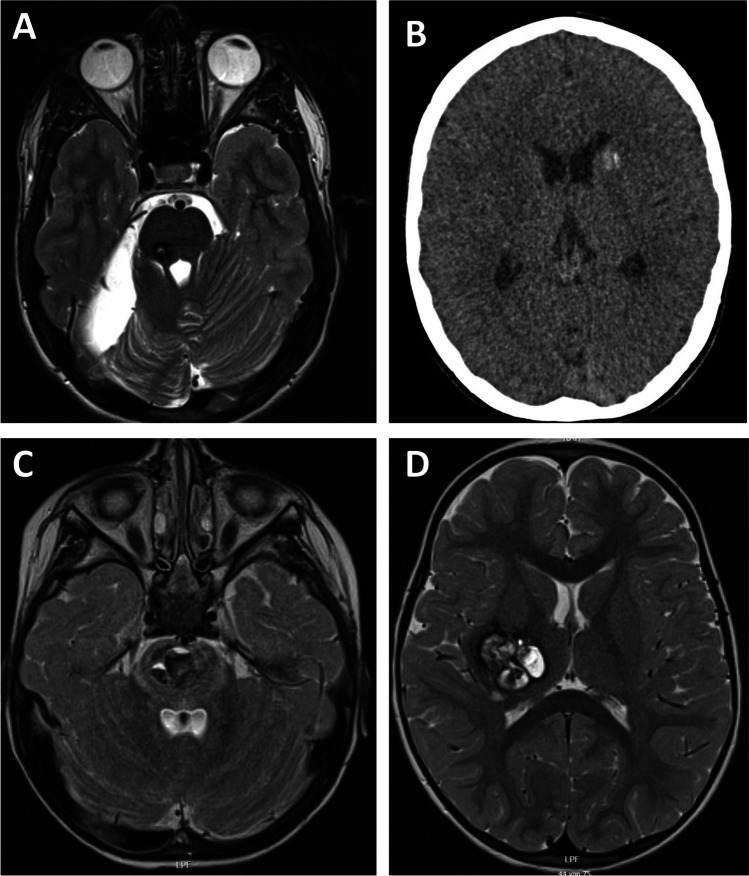
18 patients with a CM located either in the brainstem or basal ganglia. Lesions presented as smaller CMs were associated with very mild or no symptoms (**A**, **B**) while larger lesions correlated with neurological deficit (**C**, **D**)

There were 18 patients with a CM located either in the brainstem or basal ganglia. Lesions presented as smaller CMs were associated with very mild or no symptoms (Fig. 3A and B), while larger lesions correlated with neurological deficit (Fig. 3C and D). Of the whole subgroup, 78% (14/18) of patients underwent surgery and 22% (4/18) were managed conservatively. As observed in lobar lesions, patients in the surgery group presented significantly more often with symptoms of acute hemorrhage (*p* = 0.006). A total of 50% (7/14) of all patients in the surgery group received more than one surgery. The most common reason for repeat surgery was symptomatic hemorrhage, which occurred after first surgery, as described below. Within the surgical group, the median mRS at diagnosis was significantly higher than in the conservative group with (4 [2–5] vs 1 [0–2], *p* = 0.005). The most common symptom recorded at diagnosis was hemiparesis: 64% in the surgery group versus 0% in the conservative group. The median mRS recorded at time of diagnosis significantly improved to lower values at last follow-up in both the surgery and the conservative group (4 to 1 vs 1 to 0, *p* = 0.004 vs *p* = 0.083). Two patients in the surgery group had a mRS score > 2. One patient with a mRS of 4 exhibited persistent severe hemiparesis, which was already present at diagnosis and had not resolved at last follow-up. Another patient with a mRS of 3 recovered well from a pons CM; however, due to multiple CMs located in the region of the motor cortex, mRS declined during follow-up. Lesions were significantly larger in the surgery group (*p* = 0.004), and there was no lesion reaching a diameter of 1 cm in the conservative group. The postoperative complication rate was 14% in the subgroup of deep CMs. One patient suffered from worsening of a hemiparesis after surgery, which, however, resolved during the observation period, and the patient reached mRS 1 at last follow-up. Another patient suffered from a subgaleal pseudomeningocele, which resolved after revision surgery. No permanent new neurological deficits were encountered after surgery. Pre- and postoperative data from patients with deep CMs are summarized in Table 3.

**Table 3:** CMs of the brainstem and basal ganglia

	Surgery (*n* = 14)	Conservative (*n* = 4)	*p*-value
Location			
Pons	10 (71%)	2 (50%)	*p* = 0.569
Basal ganglia	4 (29%)	2 (50%)	
Hemorrhage at time of diagnosis (%)	14 (100%)	1 (25%)	***p* = 0.005**
Seizures at diagnosis	1 (7%)	2 (50%)	*p* = 0.108
Symptoms at diagnosis			
Paresis	9 (64%)	0	*p* = 0.082
Headache	2 (14%)	1 (25%)	*p* = 0.426
Other neurological deficit	11 (85%)	2 (50%)	*p* = 0.197
Median mRS at time of diagnosis (min–max)	4 (2–5)	1 (0–2)	***p* = 0.003**
Median maximum lesion diameter (cm)	2.4 (1.0–4.2)	0.8 (0.6–0.9)	***p* = 0.004**
Depth			
Superficial	7 (50%)	0	*p* = 0.119
Profound	7 (50%)	4 (100%)	
Number of interventions			
1	7 (54%)	n.a	**–**
2	5 (30%)		
3	1 (8%)		
6	1 (8%)		
Postoperative complication	2 (14%)	n.a	**–**
Gross-total resection	9 (64%)	n.a	**–**
Outcome	Surgery (*n* = 10)	Conservative (*n* = 4)	
Median time of follow-up (months)	88.0 (39–356)	59.5 (1–174)	*p* = 0.480
Postoperative median mRS	1 (0–4)	0 (0–1)	*p* = 0.101
Seizure-free at last follow-up	10 (100%)	4 (100%)	**–**
Hemorrhage during follow-up	4 (40%)	0	*p* = 0.210

Table 3 shows characteristics of surgically (*n* = 14) and conservatively (*n* = 4) treated patients with CMs located in the brain stem or basal ganglia. Surgical data for the surgery group and outcome data for surgically (*n* = 10) and conservatively (*n* = 4) treated patients is included. The treatment group differed in mRS at diagnosis, which was mainly driven by paresis as main symptom. At time of last follow-up, mRS scores of both groups aligned. Bold font indicates significance.

### Hemorrhage analysis

**Fig. 4:**
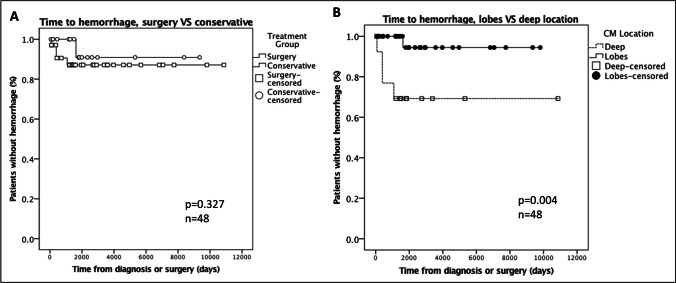
Overall risk of hemorrhage as visualized per Kaplan–Meier plot is shown in **A**. As shown in **B**, risk of hemorrhage was significantly higher in patients with deep lesions compared with patients with lobar CM

For hemorrhage analysis, data were available in 85% (52/61) of patients. Overall, one of 34 patients (3%) with lobar lesions suffered from hemorrhage during follow-up of 255 patient-years. The patient suffered from seizure recurrence after initial seizure-freedom for 4.5 years after diagnosis. At admission, computed tomography revealed hemorrhage of the CM in postcentral location. The patient was treated conservatively and AEDs were re-established. A total of 29% (4/14) of patients with deep CMs suffered from symptomatic hemorrhage during follow-up of 130 patient-years, all of which were in the surgery group and experienced worsening of symptoms at time of hemorrhage. However, repeat of surgery led to improvement of symptoms in these patients; one of these patients experienced 3 symptomatic hemorrhages of a pons CM, requiring 3 repeats of surgery. In one patient, three hemorrhages were documented during initial follow-up before a second surgery was performed. Two of the patients reached a mRS of 1 and two reached a mRS of 2 at time of last follow-up. Overall risk of hemorrhage as visualized per Kaplan–Meier plot is shown in Fig. 4A. As shown in Fig. 4B, risk of hemorrhage was significantly higher in patients with deep lesions compared with patients with lobar CM (*p* = 0.004). The calculated hemorrhage rates per patient year were 6.2% for deep CMs and 0.4% for lobar lesions.

### Treatment algorithm

**Fig. 5:**
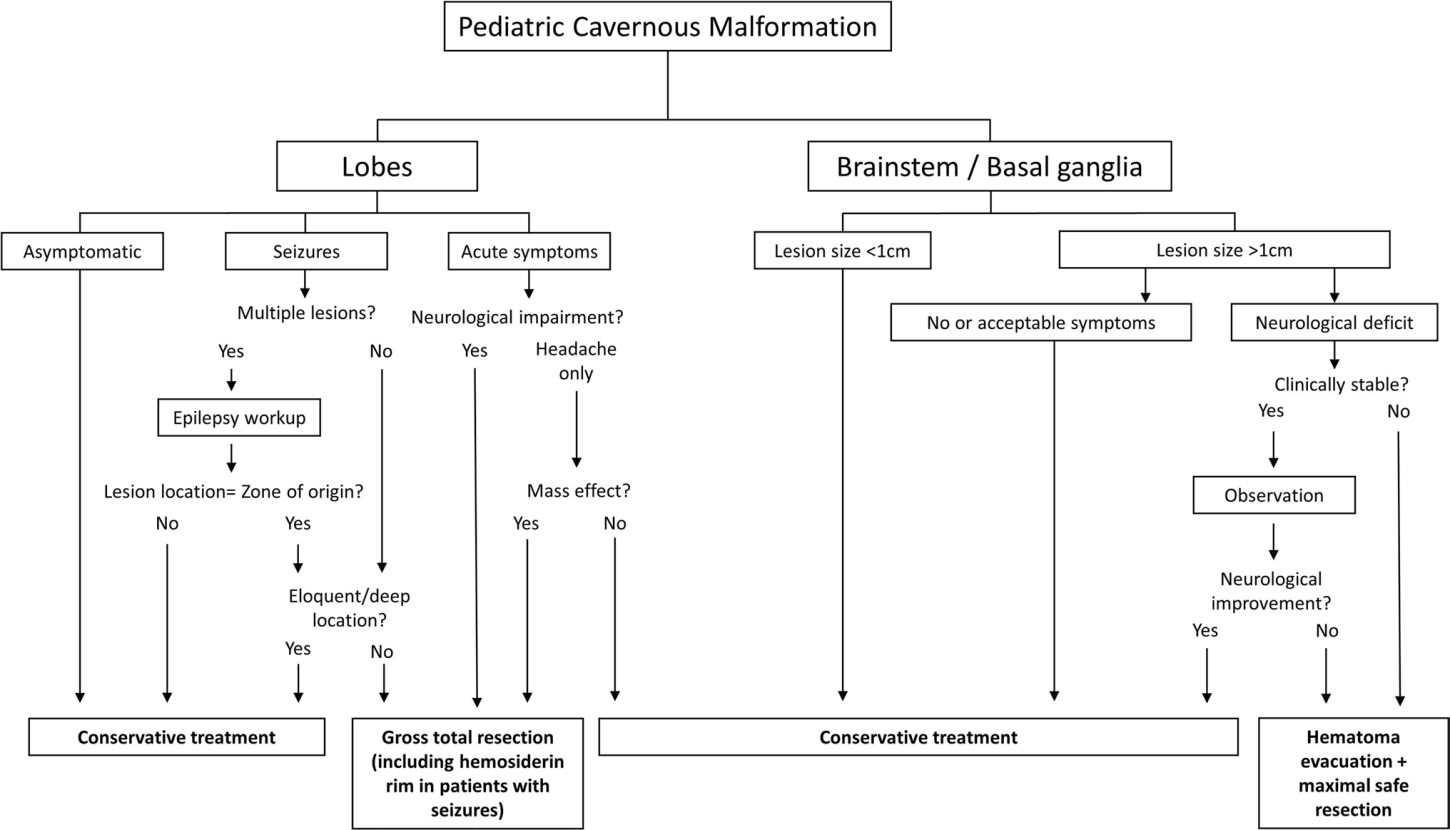
Overview of the proposed treatment algorithm that is based on our data

An overview of the proposed treatment algorithm that is based on our data is shown in Fig. 5. First, differentiation between lesions located in the lobes or in the brainstem/basal ganglia is crucial. Further treatment of lobar CMs primarily depends on results of epilepsy workup in patients with seizures and presence of acute symptoms in non-epilepsy patients. In lesions located in the brainstem or basal ganglia, the treatment approach depends on lesion size, burden of symptoms, and clinical course. Therefore, conservative treatment of CMs with a maximum diameter of < 1 cm appears to be safe. In patients with larger lesions, hematoma evacuation and maximal safe resection are indicated in cases with neurological deficits that do not resolve during observation or in cases that exhibit clinical instability.

## Discussion

Our analysis suggests that our institutional policy in the management of pediatric CM results in improved long-term outcome in these patients. Our data indicates that (i) seizure control requires extended resection of the cavernomas beyond pure lesionectomy, (ii) it is safe to manage asymptomatic lobar and small (< 1 cm) deep CMs conservatively as no patient in our study experienced a severe event, and (iii) less aggressive surgery in deep CM may require a repeat of surgery due to re-hemorrhage and symptom recurrence, but an excellent long-term neurological outcome can be expected. Based on this experience, we propose a treatment algorithm to guide clinical decision-making in the treatment of children with CM.

Even though there are some reports that are comparable to our cohort in terms of age, gender distribution, clinical presentation, and number of lesions, our data on the conservatively treated children is rather unique [1, 8, 24, 32]. While a small number of studies did include a conservative group in comparing the clinical outcome, Velz et al., for instance, exclusively reported on brain stem CMs, and in two other studies, the number of conservatively treated patients was comparably small with only 13 and 6 patients, respectively [5, 24, 30]. Such an analysis, however, is essential to understand the natural history of these lesions and enables adequate treatment allocation.

Since baseline characteristics differ significantly between groups, the goal of comparing surgically and non-surgically treated groups is not to determine whether either of these strategies leads to better results, but to compare the postoperative clinical progress to the natural course of the disease. According to our analysis, mRS scores significantly improved from time of diagnosis to time of last follow-up in both treatment groups. Furthermore, the difference in median mRS at the time of diagnosis between groups dissolved during the observation period. This finding not only indicates that surgery in patients assigned to an intervention is beneficial but also confirms the adequate selection for conservative treatment at our institution.

### Lobar lesions

CMs with lobar locality predominately present with seizures and surgery aims to achieve seizure freedom in case of pharmaco-resistance. Only severe hemorrhage due to a cavernoma necessitates urgent surgical removal [21]. In our cohort, 72% of patients had seizures at the time of diagnosis, and seizure freedom was achieved in 86% of these patients. However, 2 patients required a second surgery due to seizure recurrence after initial postoperative seizure freedom for 1 and 3.5 years, respectively. Both underwent second epilepsy surgery and were seizure-free thereafter. In both cases, the resected CM was located adjacent to an eloquent region (motor cortex and visual cortex), and aggressive resection was avoided initially. Since no permanent deficits were encountered after the second more extended surgery, an initially more aggressive approach may have been justified in these cases, which may have avoided seizure recurrence and the need for a second surgery. Therefore, a more restrained approach may not be successful if patients with lobar CM and epilepsy are allocated to a surgical treatment. Furthermore, a conservative treatment may lead to a good seizure control at a first glance, but recurrence and new-onset seizures may be encountered during the observation period. In our study population, one patient encountered seizure recurrence 1 year after cessation of AEDs, and one additional patient experienced new-onset seizures during the observation period. In both cases, AEDs were (re)established.

The postoperative seizure rates in pediatric patients with CMs reported in previous studies are diverse. For instance, a multi-center study conducted by Hugelshofer et al. that included 41 pediatric patients with CM and seizures found postoperative rates of 72% Engel I and 11% Engel II [12]. Sawarkar et al. reported seizure outcomes of 94.1% Engel I and 5.9% Engel II in a cohort including 19 patients with seizures [24]. Whereas Hugelshofer et al. reported gross total resection including the hemosiderin rim in all patients with seizures, Sawakar et al. reported resection of the hemosiderin rim only in 41% of the cases. Other studies reported 100% postoperative seizure-free rates after CM resection in pediatric patients. However, those studies by Xia et al. and Di Rocco et al. did not use a formal seizure classification, nor did they define the term “seizure-free” [8, 32]. Despite the limited number of studies that assessed seizure outcome after CM resection in pediatric patients, numerous studies focusing on adult CM patients reported findings similar to our results, suggesting postoperative seizure-free rates of approximately 75% [23]. Thus, differences in reported rates of seizure-free patients may depend on other factors than extent of resection of hemosiderin and may be explained by differences in time of follow-up or different policies in the use of AEDs.

This excellent epilepsy outcome in surgically treated patients with lobar CM can be discussed in the context of the risk for hemorrhage and operative complication rate. In our study population, only one patient in the conservative group suffered from symptomatic hemorrhage. Thus, the observed annual hemorrhage rate in the subgroup of patients with lobar lesions was 0.4%. Annual hemorrhage rates of pediatric lobar CMs reported in the literature range between 0.5 and 2.1% [10]. Also, in the adult population, conservatively managed, asymptomatic patients are at low risk of hemorrhage (3.8% in 5 years) according to a systematic review by Fleming and Lanzino [9]. Regarding the operative morbidity, we encountered no new permanent deficit with an overall complication rate of 8%. These findings are in line with previous reports and further support the utilization of our approach [8, 12]. The case of a patient who suffered from seizures and underwent resection of a lobar CM with an extensive hemosiderin deposit is described below.

### Case presentation

**Fig. 6:**
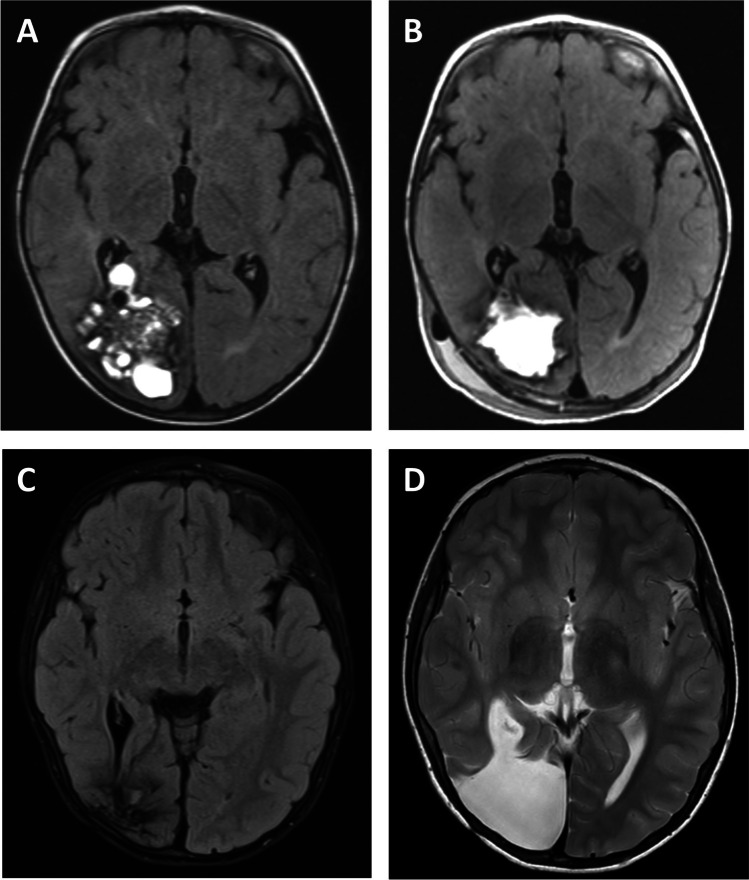
In a male 1.5-year-old patient who was exhibiting seizures, a large right occipital CM was resected adjacent to the area of the visual cortex (**A**). As shown in **B**, resection was restricted to the CM itself without radical removal of the hemosiderin rim. Follow-up MRI showed persisting hemosiderin deposits in the cortex around the resection cavity (**C**). After extension of the initial resection, which included all hemosiderin remnants, the patient achieved seizure-freedom (**D**)

In a male 1.5-year-old patient who was exhibiting seizures, a large right occipital CM was resected adjacent to the area of the visual cortex (Fig. 6A). As shown in Fig. 6B, resection was restricted to the CM itself without radical removal of the hemosiderin rim. After surgery, the patient was seizure-free with seizure recurrence observed 3.5 years after the surgical intervention. Follow-up MRI showed persisting hemosiderin deposits in the cortex around the resection cavity (Fig. 6C). Video EEG monitoring confirmed localization of the epileptogenic zone in the field of resection. After extension of the initial resection, which included all hemosiderin remnants, the patient achieved seizure-freedom (Fig. 6D).

### Lesions of the brainstem and basal ganglia

The current study assessed 18 children with CMs of the brainstem or basal ganglia with the majority (78%) receiving surgery. The decision for a conservative treatment in the remaining 4 children was based on the low symptom burden, small lesion size, and difficult surgical accessibility of the lesion. No hemorrhage was seen in any of the conservatively managed patients (median follow-up time: 59.5 months). Although deep location is reported to be a risk factor for hemorrhage, lesion size is likely to be crucial [10, 21]. According to a study by Li et al., which included 85 pediatric patients with untreated brainstem CMs, a lesion diameter of > 2 cm is associated with higher hemorrhage risk [16]. An association between size and hemorrhage risk has also been reported by Kupersmith et al. in a series of 37 adult brainstem CMs with a cut-off value of 10 mm [14]. In our cohort, conservatively managed CMs had a median diameter of 0.8 cm (0.6–0.9 cm) compared with a median diameter of 2.4 cm (1.0–4.2 cm) in surgically treated patients. Consequently, the observed benign course of the conservative group may be attributable to the significantly smaller lesion size.

The surgically treated group of patients was characterized by neurological impairment at the time of presentation. Hemiparesis was the leading neurological deficit followed by other symptoms such as reduced state of consciousness and cranial nerve palsies. Significant improvement of the median mRS score from 4 to 1 in the surgery group confirms the substantial benefit from surgery in this group. Gross-total resection after last surgery was achieved in 64% of patients with no newly encountered permanent neurological deficit. This is in marked contrast to other reports. Abla et al., for instance, reported a rate of 25% of permanent new deficits after surgery in 40 pediatric patients with brainstem CMs [1]. These findings are in line with a study by Li et al., who reported 21.2% permanent new deficits in a cohort of 52 children with brainstem CMs [17]. The discrepancy between our and previous findings is likely due to the fundamentally different treatment approach. While it has been our policy to be less aggressive, leaving residual disease in place before putting the patients at risk for permanent neurological compromise, and to re-operate in case of recurrence, others share the opinion that total removal is necessary irrespective of the potential morbidity during surgery [5, 17]. These two treatment concepts can be discussed under various aspects; the fact, however, that no child with deep CM in our study population experienced neurological compromise due to re-hemorrhage in residual disease or multiple surgeries strongly favors the less aggressive approach utilized in this study. This difference in conceptualizing surgery for deep CM is confirmed when rates of total resection are compared. Li et al. who employed a more aggressive approach reported a gross-total resection rate of 94.2% compared with 69% gross-total resection rate after last intervention in our study [17]. Although Abla et al. did not provide information on gross-total resection rates, the difference in surgical approaches may be extrapolated by comparing the numbers of procedures with our cohort. Only 5% of patients underwent re-operation in the cohort assessed by Abla and colleagues, whereas almost half of the patients in our study population received at least two surgical procedures [1]. The main argument for an aggressive approach has been advocated to be the potential for re-hemorrhage. According to the study by Li et al., the annual hemorrhage rate declined from 12.3 to 0.5% after surgery [17]. However, Abla et al. reported an annual postoperative hemorrhage rate of 5.25% versus 6.2% revealed during follow-up in the current analysis [1]. Applying these findings to a corresponding clinical situation in which patients, parents, and surgeons are diversely involved with regard to their understanding of the medical issue, the decision about the intended extent of resection must be an individual and very personalized one. The data in our study suggest that a cautious approach, not primarily aiming at gross total resection, results in a clear benefit for patients with a relatively small complication rate and no permanent new neurological deficit. One reason for the observed favorable neurological outcome even in patients who suffer from re-hemorrhage after partial resection might be the following hypothetic mechanism: In case of re-hemorrhage, the cavity which was created through partial CM resection may reduce the potential mass effect of a low-pressure bleeding by providing a pre-formed space, resulting in a lesser degree of displacement or destruction of the parenchyma. Our policy is further supported by the exemplary case described below.

### Case presentation

**Fig. 7:**
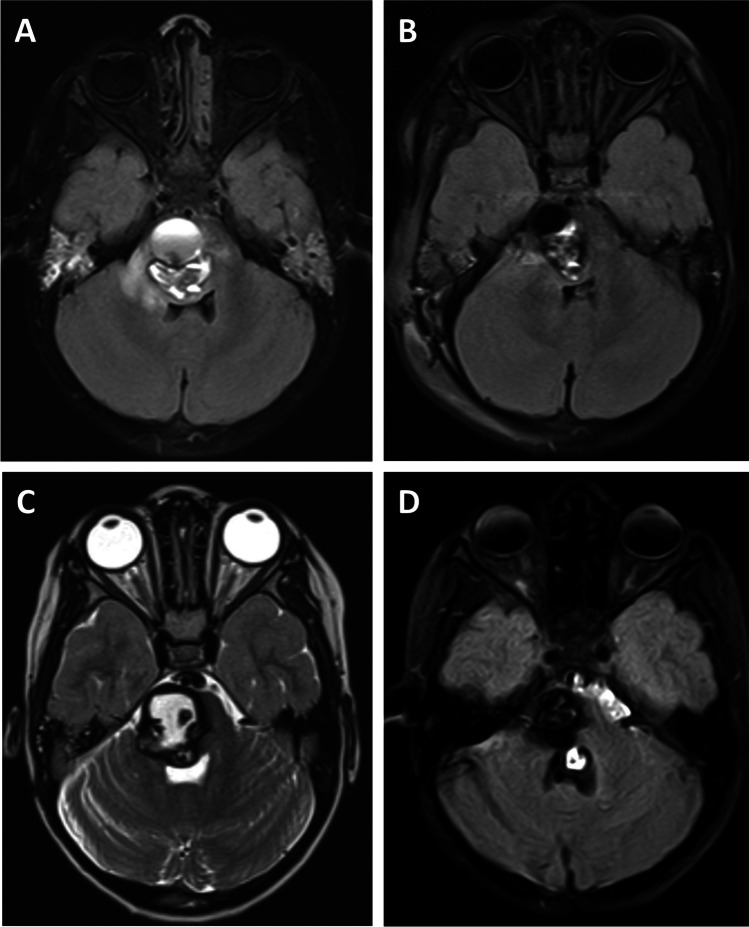
3-year-old female patient with symptomatic hemorrhage of a large CM located in the pons (**A**). After hematoma evacuation and partial resection of the CM via a retrosigmoidal approach (**B**), the patient recovered and developed well. One year later, asymptomatic progression was seen during a routine follow-up MRI (**C**). Although the lesion was still evident during a follow-up MRI almost 4 years later (**D**), the patient experienced no further neurological symptoms since the last surgical intervention

A 3-year-old female patient with symptomatic hemorrhage of a large CM located in the pons (Fig. 7A) presented with VI nerve palsy and mild hemiparesis. After hematoma evacuation and partial resection of the CM via a retrosigmoidal approach (Fig. 7B), the patient recovered and developed well. One year later, asymptomatic progression was seen during a routine follow-up MRI (Fig. 7C). For this reason, an elective surgery was performed, using an approach via the telovelar route. Given that no safe corridor could be identified based on the findings from neuromonitoring without putting the geniculum of the facial nerve or the medial lemniscus pathway at risk, no resection was performed. Although the lesion was still evident during a follow-up MRI almost 4 years later (Fig. 7D), the patient experienced no further neurological symptoms since the last surgical intervention.

### Limitations

Limitations of the current study are due to its retrospective design and involve the potential influence of unmeasured variables and confounding factors. Baseline characteristics between the conservative and surgical treatment group differ significantly in parts, which makes a direct comparison difficult. Moreover, the cohort of conservatively managed patients with lesions in deep, eloquent regions includes only a small number of patients, resulting in limited comparability, and lesions located in the brainstem and basal ganglia were pooled into one group. This may affect comparability with other cohorts. However, we regard CMs located in deep-seated locations as an exclusive surgical entity since total resection is usually much more limited than in other regions, and severity of symptoms is similar.

## Conclusions

A conservative management in pediatric patients with asymptomatic CM is safe. In patients with symptomatic lobar CM, gross total resection should be the aim. In deep CM, a less aggressive approach with subtotal resection (if deemed necessary to avoid neurological compromise) leads to an improved and sustained neurological outcome.

## Data Availability

Not applicable.
